# Complete Genome Sequence of *Vibrio anguillarum* M3, a Serotype O1 Strain Isolated from Japanese Flounder in China

**DOI:** 10.1128/genomeA.00769-13

**Published:** 2013-09-26

**Authors:** Guiyang Li, Zhaolan Mo, Jie Li, Peng Xiao, Bin Hao

**Affiliations:** Laboratory of Maricultural Organism Disease Control and Molecular Pathology, Yellow Sea Fishery Research Institute, Chinese Academy of Fishery Sciences, Qingdao, Chinaa; Key Laboratory of Experimental Marine Biology, Institute of Oceanology, Chinese Academy of Sciences, Qingdao, Chinab; State Key Laboratory of Microbial Resources, Institute of Microbiology, Chinese Academy of Sciences, Beijing, Chinac

## Abstract

*Vibrio anguillarum* is an important bacterial pathogen that causes vibriosis in marine fish. We present the complete genome sequence of *V. anguillarum* M3, a serotype O1 clinical strain isolated from Japanese flounder (*Paralichthys olivaceus*) in Shandong, China.

## GENOME ANNOUNCEMENT

*Vibrio anguillarum* causes vibriosis, a systemic disease of both wild and cultured marine fish characterized by hemorrhagic septicemia (1). There are 23 serotypes identified for this bacterium, and the O1 and O2 serotypes are the major causative agents of fish vibriosis (2). Recently, the genome sequences of three *V. anguillarum* strains, isolated from salmon (*Oncorhynchus kisutch*), striped bass (*Morone saxatilis*), and turbot (*Scophthalmus maximus*), were released (3). Here, we present the complete genome sequence of *V. anguillarum* M3, an O1 serotype clinical strain isolated from Japanese flounder (*Paralichthys olivaceus*) (4). This strain has been studied with respect to its *in vivo* antigen gene expression, metalloprotease and exopolysaccharide production, and biofilm formation (5–7).

The genome of *V. anguillarum* M3 was sequenced using the Roche 454 GS FLX Titanium system at SeqWrite, Inc. (Houston, TX). A total of 23,745 reads were obtained from the sequencing (~20-fold coverage of the genome). The reads were assembled using DataAnalysis version 2.6 (Roche/454 Life Sciences), generating 59 contigs. Using the genome of *V. anguillarum* strain 775 as a reference, all gaps were filled by local assembly using the CLC Genomics Workbench (64-bit) 6.0.5 and ABI 3730 sequencing of the PCR products of the gap region. The coding sequences (CDSs) were predicted by the NCBI Prokaryotic Genomes Automatic Annotation Pipeline (PGAAP) and the Rapid Annotations using Subsystems Technology (RAST) server (8). Noncoding RNAs were identified using tRNAscan-SE 1.21 (9) and RNAmmer 1.2 (10) by searching against the Rfam database (11). Functional assignment of the CDSs was performed on the InterProScan (12) and the Swiss-Prot and trEMBL databases (13).

The complete genome of *V. anguillarum* M3 contains two circular chromosomes and a plasmid. Chromosome 1 has 3,063,587 bp containing 2,842 CDSs, chromosome 2 has 988,134 bp containing 918 CDSs, and the plasmid has 66,164 bp containing 64 CDSs. Overall, the M3 and 775 genomes share high levels of similarity, as >90% of the CDSs have the same annotation, length, and relative position. With respect to potential virulence traits, a majority of the virulence genes are conserved in both strains, such as those for hemolysins, metalloproteases, repeat in toxin (RTX) toxins, and vibriolysin (14). Both strains present two type VI secretion system loci on chromosome 1 and chromosome 2, and the genes in the two loci are organized identically in M3 but differently in 775 (15). In addition, M3 has 74 specific genes, encoding a transposase and 73 hypothetical proteins; 775 has 168 specific genes, including genes encoding mobile element proteins (30 genes), molybdopterin biosynthesis proteins (MoeA-MoeB), dGTPase, methyl-accepting chemotaxis protein, tryptophanase, and ribonuclease E inhibitor RraB. Regarding the pathogenicity of M3, the complete genome sequence of this strain will increase the understanding of the evolution of *V. anguillarum* and will contribute new insights into virulence studies of this particular species.

### Nucleotide sequence accession numbers.

The sequence and annotation of the *V. anguillarum* M3 genome have been deposited in GenBank under accession no. CP006699 (M3 chromosome 1), CP006700 (M3 chromosome 2), and CP006701 (M3 plasmid).

## References

[1] Austin B Austin DA 2007 Bacterial fish pathogens: disease of farmed and wild fish. Springer Verlag, New York, NY

[2] Larsen JL Pedersen K Dalsgaard I 1994 *Vibrio anguillarum* serovars associated with vibriosis in fish. J. Fish Dis. 17:259–267

[3] Naka H Dias GM Thompson CC Dubay C Thompson FL Crosa JH 2011 Complete genome sequence of the marine fish pathogen *Vibrio anguillarum* harboring the pJM1 virulence plasmid and genomic comparison with other virulent strains of *V. anguillarum* and *V. ordalii*. Infect. Immun. 79:2889–2900 21576332 10.1128/IAI.05138-11 PMC3191993

[4] Mo ZL Tan XG Xu YL Zhang PJ 2001 A *Vibrio anguillarum* strain associated with skin ulcer on cultured flounder, *Paralichthys olivaceus*. Chin. J. Oceanol. Limnol. 19:319–326

[5] Zou YX Mo ZL Hao B Ye XH Guo DS Zhang PJ 2010 Screening of genes expressed *in vivo* after infection by *Vibrio anguillarum* M3. Lett. Appl. Microbiol. 51:564–569 20849396 10.1111/j.1472-765X.2010.02935.x

[6] Mo ZL Guo DS Mao YX Ye XH Zou YX Xiao P 2010 Identification and characterization of *prtV* gene in *Vibrio anguillarum*. Chin. J. Oceanol. Limnol. 28:55–61

[7] Hao B Mo ZL Xiao P Pan HJ Lan X Li GY 2013 Role of alternative sigma factor 54 (RpoN) from *Vibrio anguillarum* M3 in protease secretion, exopolysaccharide production, biofilm formation, and virulence. Appl. Microbiol. Biotechnol. 97:2575–2585 22940804 10.1007/s00253-012-4372-x

[8] Aziz RK Bartels D Best AA DeJongh M Disz T Edwards RA Formsma K Gerdes S Glass EM Kubal M Meyer F Olsen GJ Olson R Osterman AL Overbeek RA McNeil LK Paarmann D Paczian T Parrello B Pusch GD Reich C Stevens R Vassieva O Vonstein V Wilke A Zagnitko O 2008 The RAST server: rapid annotations using subsystems technology. BMC Genomics 9:75. 10.1186/1471-2164-9-75 18261238 PMC2265698

[9] Lowe TM Eddy R 1997 tRNAscan-SE: a program for improved detection of transfer RNA genes in genomic sequence. Nucleic Acids Res. 25:955–964 9023104 10.1093/nar/25.5.955 PMC146525

[10] Lagesen K Hallin P Rødland EA Staerfeldt HH Rognes T Ussery DW 2007 RNAmmer: consistent and rapid annotation of ribosomal RNA genes. Nucleic Acids Res. 35:3100–3108 17452365 10.1093/nar/gkm160 PMC1888812

[11] Griffiths-Jones S Moxon S Marshall M Khanna A Eddy SR Bateman A 2005 Rfam: annotating non-coding RNAs in complete genomes. Nucleic Acids Res. 33:D121–D124. 10.1093/nar/gki081 15608160 PMC540035

[12] Hunter S Jones P Mitchell A Apweiler R Attwood TK Bateman A Bernard T Binns D Bork P Burge S de Castro E Coggill P Corbett M Das U Daugherty L Duquenne L Finn RD Fraser M Gough J Haft D Hulo N Kahn D Kelly E Letunic I Lonsdale D Lopez R Madera M Maslen J McAnulla C McDowall J McMenamin C Mi H Mutowo-Muellenet P Mulder N Natale D Orengo C Pesseat S Punta M Quinn AF Rivoire C Sangrador-Vegas A Selengut JD Sigrist CJ Scheremetjew M Tate J Thimmajanarthanan M Thomas PD Wu CH Yeats C Yong SY 2012 InterPro in 2011: new developments in the family and domain prediction database. Nucleic Acids Res. 40:D306–D312. 10.1093/nar/gkr948 22096229 PMC3245097

[13] Magrane M UniProt Consortium 2011 UniProt Knowledgebase: a hub of integrated protein data. Database (Oxford) 2011:bar009. 10.1093/database/bar009 21447597 PMC3070428

[14] Naka H Crosa JH 2011 Genetic determinants of virulence in the marine fish pathogen *Vibrio anguillarum*. Fish Pathol. 46:1–10 21625345 10.3147/jsfp.46.1 PMC3103123

[15] Weber B Hasic M Chen C Wai SN Milton DL 2009 Type VI secretion modulates quorum sensing and stress response in *Vibrio anguillarum*. Environ. Microbiol. 11:3018–3028 19624706 10.1111/j.1462-2920.2009.02005.x

